# Technological and Sensory Aspects of Macaroni with Free or Encapsulated *Azolla* Fern Powder

**DOI:** 10.3390/foods11050707

**Published:** 2022-02-27

**Authors:** Essam Mohamed Elsebaie, Galila Ali Asker, Mona Metwally Mousa, Mona Morgan Kassem, Rowida Younis Essa

**Affiliations:** 1 Food Technology Department, Faculty of Agriculture, Kafrelsheikh University, Kafr El-Shiekh 33516, Egypt; rowida.eisa@agr.kfs.edu.eg; 2 Food Science & Technology Department, Faculty of Home Economics, Al-Azhar University, Tanta 31511, Egypt; abdelgaleelmo@gmail.com (G.A.A.); baraahmebara@gmail.com (M.M.M.); 3 Agricultural Engineering Department, Faculty of Agriculture, Kafrelsheikh University, Kafr El-Shiekh 33516, Egypt; mona2kassem@gmail.com

**Keywords:** FAP, antioxidant activities, cooking quality, encapsulation

## Abstract

*Azolla* might be considered an alternative and promising dietary ingredient for antioxidants. There have not been any reports on the incorporation of free *Azolla* fern powder (FAP) or its microcapsules in foods, especially fresh pasta, yet. Microencapsulation was used to mask the undesirable taste and odour of *Azolla,* as well as to preserve its antioxidant potential. The current study concentrated on two major goals. The first goal was to use alginate as a wall material for FAP encapsulation, as well as to characterise the FAP microcapsule for its encapsulation efficiency, solubility, and thermal stability. The second goal was to assess the impact of integrating FAP or its microcapsules into fresh macaroni on its colour parameters, cooking quality, texture properties, and sensory characteristics. The microspheres had a high encapsulation efficiency (88.19%) and a low water solubility (85.23 g/kg), making them suitable for use in foods that require cooking in water. When compared to free *Azolla* powder, encapsulation reduced the antioxidant activity loss rate by 67.73%. All the cooking and textural properties of fresh macaroni were not significantly affected, except for water absorption and weight gain, but the overall acceptability index (85.13%) was not affected by microcapsule incorporation.

## 1. Introduction

*Azolla* (*Azolla pinnata*) is a fresh water fern that belongs to the *Azollaceae* family and the order Pteridophyta. *Azolla* is made up of six different species. It is usually grown in the tropic and sub-tropical regions. It is naturally found in the standing water of canals, drains, ponds, wetlands, rivers, and marshy regions [1]. *Azolla* fern has several uses, such as medicine, human food, and animal food [2]. *Azolla* fern has a high protein level, with a total protein value of 25–30%. Other compounds that are found in *Azolla* are vitamins, carotenoids, minerals, amino acids, chlorophyll, etc. It is also thought to be an excellent source of natural antioxidants [3]. The most abundant phenolic compounds identified in *Azolla* include anthocyanidins, Quercetin, coumarins, flavonols, flavones, and condensed tannins [4,5,6,7,8].

Macaroni is an ancient cereal-based food that dates back to the first century BC [9]. Macaroni is popular all around the world, even in developing countries, due to its low cost, simplicity of preparation, variety, sensory qualities, and extended shelf life [10]. According to current trends, the consumption of fortified foods has been increasingly popular in recent years. Hence, due to the fact that macaroni is a basic meal that can easily be fortified with non-traditional components, particular macaroni products have been developed that include additives that improve protein content while also providing useful characteristics, such as fibre and antioxidants [11]. In fact, pasta has already been enriched with several natural ingredients for improving its biological activity, such as green barley powder [12], black locust flower [13], mushroom powder [14], grape marc powder [15], and microalgal biomass [16,17,18,19]. However, the fortification of foods with algae in general faces two main problems, namely the appearance of an undesirable taste and flavour, in addition to the lack of thermal stability of its antioxidants, especially in the case of foods that require heat when preparing, such as fresh macaroni [20].

*Azolla*’s antioxidants in macaroni are protected by microencapsulation, which employs water-insoluble shell substances, due to the pasta’s cooking in hot water. The most extensively used encapsulating method is alginate gelation, which involves dropping a solution of sodium alginate and bioactive components into a calcium chloride gelation bath [21].

There have not been any reports on the incorporation of FAP or its microcapsules in foods, especially fresh macaroni, yet. Responding to consumers requests for more nutritious and functional foods, the food industry’s challenge is to keep the functional properties of a food after cooking while preserving the product’s desired sensory appeal. In this respect, the current study concentrated on two major goals. The first goal was to use alginate as a wall material for FAP encapsulation, as well as to characterise the FAP microcapsule for its encapsulation efficiency, solubility, and thermal stability. The second goal was to assess the impact of integrating free FAP or its microcapsules into fresh macaroni on its colour parameters, cooking quality, texture properties, and sensory characteristics.

## 2. Materials and Methods

### 2.1. Materials

Fresh *Azolla* fern biomass was gathered from the aquatic greenhouse of the Egyptian Agriculture Research Center during the months of June to August of 2020. Sodium alginate (SA), Calcium Chloride, and 2,2-azinobis (3-ethylbenzothiazolin)-6-sulfonic acid (ABTS) were secured from Sigma Aldrich Co. (St. Louis, MO, USA).

Wheat flour (72% extraction) was commercially obtained from Ibn EL-Khatab Mills Company, 6th October City, Egypt. The wheat flour had a moisture content of 13.10 ± 0.03, protein content of 12.25 ± 0.07, lipid content of 0.78 ± 0.03, and ash content of 0.95 ± 0.12. The gluten index, wet, and dry gluten were 98.2 ± 0.41, 19.83 ± 0.62, and 7.6 ± 0.3 g/100 g, respectively. All the analyses were performed in triplicate in accordance with the AACC [22].

All other items used in pasta preparation were obtained from a local shop (Kafr El-Shiekh city, Egypt).

### 2.2. Methods

#### 2.2.1. Preparation of FAP

Cultivated *Azolla* fern biomass was properly rinsed in fresh water, weighed, and left to dry for 9 days in the shade at 50% relative humidity at 28 °C, resulting in crispy *Azolla* that preserved its green colour. Dried *Azolla* was gathered, put in airtight containers, and kept until needed. The dried *Azolla* was roughly ground, using a screen size of 60 mesh. At that time, the moisture content was 6.19 ± 0.07 g/100 g, ash content was 13.86 ± 0.76 g/100 g, protein content was 33.72 ± 0.98 g/100 g, fat content was 3.95 ± 0.03 g/100 g, and crude fibre content was 9.95 ± 1.13 g/100 g in the dried FAP. All analyses were performed in triplicate in accordance with AOAC [23].

#### 2.2.2. *Azolla* Encapsulation

The beads were created via the method revealed by Yan et al. [24], with slight modification. A solution of SA (1.5% *w/w*) was produced by dispersing it in deionised water and stirring it for 12 h at 24 °C using a magnetic stirrer. The FAP (37 g/L of SA solution) was added after generating a homogenous SA solution and was homogenised for 30 min. As a crosslinking agent, the mixture was dripped into 100 mL of calcium chloride (1.1% *w/w*) using a 20 G flat-tipped hypodermic needle at a distance of 30 cm. The microspheres were then filtered and rinsed three times with deionised water (300 mL) before being lyophilised (Liotop L101, Liotop Indústria de Liofilizadores, São Carlos/SP, Brazil). Glassy particles were gathered and packed in polyethylene bags before being preserved for further usage and analysis.

#### 2.2.3. Microsphere Characterisation

##### Microscopic Observations

The beads outer surfaces were observed with a JOEL6360LA (JEOL Ltd., Tokyo, Japan) scanning electron microscope at an acceleration voltage of 20 kV.

##### Encapsulation Efficiency

The quantity of FAP that remained in the microspheres was divided by the original amount of FAP supplied to each formula to calculate the encapsulation efficiency (%). According to the protein content of the microspheres and the overall protein content of FAP, the quantity of FAP left in each formulation was calculated. The protein was quantified using the Kjeldhal method [23], with a total nitrogen to crude protein conversion factor of 6.25.

(1) $$\mathrm{Encapsulation}\;\mathrm{efficiency}\;\left(\%\right)=\frac{F}{C+A}\times 100$$

where F denotes the protein content (g) per kilogramme of FAP-coated beads, C is the protein content (g) per kg of FAP, and A is the protein content (g) per kg of empty beads.

##### Solubility

Solubility was assessed using the technique reported by Cano-Chauca et al. [25], with certain changes. The goal was to see if the microcapsules were soluble and if the substance could be released from the microcapsule core during the macaroni cooking procedures. The microcapsules (0.5 g) were put in 50 mL of boiling deionised water and were cooked for 5 min at the same temperature as the macaroni cooking. The microcapsule suspension was chilled to ambient temperature before being centrifuged for 15 min at 5439× *g*. A 12.5 mL aliquot of supernatant was placed in a pre-weighed porcelain container and was oven dried (Memmert, Schwabach, Germany) for 5 h at 105 °C. The following equation was used to compute the solubility (%):

(2) $$\mathrm{Solubility}\;\left(\%\right)=\left[\;\frac{(\mathrm{solid}\;\mathrm{mass}\;\mathrm{in}\;25\;\mathrm{mL}\;\mathrm{of}\;\mathrm{solution})\times 2}{\mathrm{Mass}\;\mathrm{of}\;\mathrm{FAP}\;\mathrm{microspheres}}\;\right]\times 100$$

##### Stability of the FAP Microcapsule Antioxidant Activities

The total antioxidant activity of the investigated watery extracts was determined with ABTS as a free radical, as reported by Re et al. [26]. Different amounts of Trolox were used to create a standard curve, and the findings were represented as mmol of trolox equivalent/g *Azolla*. The watery extract was prepared as mentioned by López-Córdoba et al. [27]. For this objective, FAP (0.7 g) or FAP microcapsules (1 g) were utilised. For adding the same amount of *Azolla* fern to the macaroni formulations, the FAP microcapsule mass to be incorporated into the macaroni was determined based on the encapsulation efficiency result. FAP microcapsules or FAP were mixed into 100 mL of water and boiled at 100 °C for 5 min, which is the recommended fresh macaroni cooking time. The control experiment was carried out at room temperature. For antioxidant activity measurement, the heated and control samples were put in an orbital shaking incubator (ES-20/80) at 1580 g for 20 h at 36 °C and then exposed to triple freeze/thaw cycles, with one cycle equivalent to 3 h of freezing and 3 h of thawing. This method is required to break down the cell wall in order to extract the antioxidant components. The extracts were centrifuged for 15 min at 4910× *g*, filtrated, and tested for their antioxidant activities.

#### 2.2.4. Fresh Macaroni Production

The fresh control macaroni (CM) was made using the proportions specified by Essa and Mohamed [28]. The CM formula consisted of 100 g of wheat flour, 0.5 g of sodium chloride (NaCl), 15 g of pasteurised eggs, and 17 mL of deionised water. The formula was blended in a vertical mixing machine (model KSM150, KitchenAid, MI, USA) for 15 min, and the dough was then subjected to an extruder (DEMARCO, Mod S-25DeFrancisci, West Melbourne, FL, USA) at the Agricultural Research Centre (Giza, Egypt) through a bronze die (die hole diameter, 1.50 mm) to produce a 30-cm-long noodle shape. Macaroni formulations containing empty beads (EB), FAP, or FAP microcapsule were also prepared. The EB formula was prepared in order to assess the impact of the encapsulating shell material on macaroni properties. The *Azolla* addition amount in both the FAP and FAP microcapsule macaroni formulas was 3 g. The quantity of FAP microcapsules or empty beads was determined as a function of the encapsulation efficiency, encapsulating shell substance amount, and FDA daily ingestion limits. After making fresh macaroni, all formulae were kept in sealed containers at −40 °C until required. For all analyses, just one batch of each macaroni type was made.

#### 2.2.5. Fresh Macaroni Quality

##### Chemical Quality Indicators

The moisture content of all fresh macaroni samples was measured, as mentioned, by the AACC [22]. Water activity was measured using a relative humidity meter (hygrometer-HS2, Novasina, Switzerland) [29].

##### Microbiological Analysis

Pasta (10 g) was homogenised with 90 mL of sterile saline peptone solution and was stirred for 10 min. Specific media were utilised for microbial counts. Plate count agar (Difco Laboratories, Detroit, Michigan, USA) was used for estimating the total bacterial count after 24 h of incubation at 37 °C. The total yeast and mould count were estimated using Sabouraud’s dextrose agar media (Oxoid, Basingstoke, UK) after incubation at 25 °C for 5 days. The microbiological analysis was conducted as mentioned by APHA [30].

##### Colour Measurement

A colorimeter (CR410, Minolta Co, Osaka, Japan) was used to determine the L*, a*, and b* colour characteristics of the fresh macaroni, as mentioned by Minolta [31]. Each sample in each batch was subjected to seven measurements.

##### Fresh Macaroni Cooking Attributes

The optimal cooking time, cooking loss, water absorption index (WAI), and swelling index determinations were calculated as mentioned by the AACC [22]. Fresh macaroni (20 g) was cooked in boiling filtered water (280 mL) until the interior white core vanished, which was observed at 30 s intervals to determine the optimum cooking time. The macaroni was rinsed for 1 min after cooking to remove any remaining water and was dried for 3 h at 105 °C until it reached a consistent weigh. The cooking water was dried at 105 °C for 3 h until it reached a consistent weight to determine the solids lost from the macaroni. Cooking loss was measured as grams of substance lost in cooking water per 100 g of uncooked macaroni. The following equations were used to calculate the WAI and swelling index:

(3) $$\mathrm{WAI}\left(\%\right)=\frac{W0-W}{W}\times 100$$

(4) $$\mathrm{Swelling}\;\mathrm{index}\;\left(\%\right)=\frac{W0-\mathrm{WD}}{\mathrm{WD}}\times 100$$

where: W refers to the weight of raw macaroni (in grams), W0 is the weight of cooked macaroni (in grams), and WD demonstrates the weight of cooked macaroni after drying at 105 °C (in grams).

##### Texture

The cooked macaroni firmness was assessed using the HDP/LKB probe at a 0.17 mm/s velocity and a 4.5 mm distance from the samples in the TA.XT Plus Texture Analyzer (Stable Micro Systems, UK). The HDP/PFS probe was used to assess the cooked macaroni stickiness at a (0.50 mm/s) speed and a (5 mm) distance away from the sample according to the AACC [22]. Each sample in each batch was subjected to five measurements.

##### Sensory Evaluation

Twenty trained panellists from Kafrelshiekh University’s Food Technology Department in the Agriculture Faculty were invited for the testing. The experiment took place in an area of the lab that was lit by white fluorescent lights. For each judge, 20 g of each fresh macaroni formulation was cooked for 5 min in separate vessels. Individually prepared macaroni was salted (0.5% sodium salt) and served without sauces in a plastic container with four-digit random numbers in a regulated presentation order. The panellists evaluated the macaroni’s colour, taste, texture, odour, and overall acceptability. For each characteristic analysed, a 9 point hedonic scale ranging from 9 (like extremely) to 1 (dislike excessively) was used [32]. The accepted samples were those that had an acceptability index of 70% or more (determined by the mean of overall acceptability/9 × 100) [33].

#### 2.2.6. Statistical Analysis

All measurements were carried out in triplicate, according to Steel and Torrie [34]. The obtained results were analysed by a one-way ANOVA (*p* ≤ 0.05) in the general linear model of SPSS (Ver. 16.0, 2007, IBM, Armonk, NY, USA).

## 3. Results and Discussion

### 3.1. Microsphere Characterisation

#### 3.1.1. Microscopic Observations

**Figure 1:**
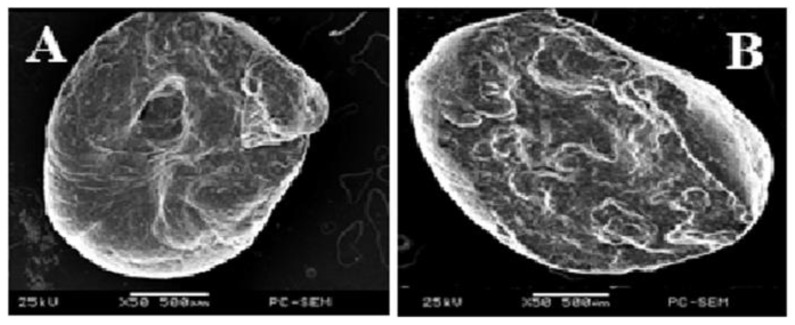
SEM image of alginate beads with encapsulated *Azolla* fern powder, showing the outer aspect (**A**) and cross-section (**B**).

The SEM images in Figure 1A,B) reveal that the formed microcapsules were spherical. The existence of diverse exterior morphologies (smooth and rough surfaces) was detected as a result of drying and subsequent shrinking, according to surface observations (Figure 1A). The cross-section of these beads containing FAP (Figure 1B) revealed that they had the structure of a three-dimensional porous sponge made of alginate. Our findings were consistent with what was stated by Belščak-Cvitanović et al. [35]. They observed a rough and nonhomogeneous surface of alginate-protein beads, but they delegated the spherical constructions to proteins, stating that morphologies are ascertained by the covalent binding between the amide groups of the protein and alginate.

#### 3.1.2. Encapsulation Efficiency (%) and Solubility

Alginate beads have been criticised for high gel porosity, which allows loaded bioactive components to diffuse from the gel network into the aqueous medium. In our study, an encapsulation efficiency of 88.19 ± 1.08% was attained, indicating that the approach utilised for encapsulation enabled the substantial retention of FAP in the calcium-alginate matrix while affecting the production of regular spherical microcapsules, as seen in the microscopic examination. The attainment of a high encapsulation efficiency allows us to acquire the necessary concentrations of active substances in various food items by incorporating a small quantity of microparticles. It is also interesting to mention that lower microcapsule quantities added to formulations encourage the keeping of sensory properties of food items, such as flavour and texture [36].

The FAP microcapsules had a water solubility of 8.52 ± 1.04%, making them perfect for inclusion into fresh macaroni because the macaroni structure helped protect these microcapsules, assuring no degradation of the core substance in the structure throughout baking.

#### 3.1.3. Stability versus Water Heat Treatment

When heat treatment was applied to FAP, it lost 36.57% of its antioxidant activity (Table 1), demonstrating that the antioxidant contained in FAP is thermo-unstable. When compared to FAP, the FAP microcapsules exhibited a loss rate of 11.80% in antioxidant activity; hence, the encapsulation reduced the antioxidant activity loss rate by 67.73%.

Furthermore, we hypothesised that adding microcapsules to the macaroni would result in an extra protective impact on the antioxidant activities, owing to the existence of the food matrix. As a result, we determined that the selected microencapsulation approach was sufficient for protecting FAP antioxidants for subsequent incorporation into food that needs cooking in water. Table 1 further demonstrates that under non-heating conditions, the FAP antioxidant activity was significantly greater than that of FAP microcapsules owing to the resistance to breakdown of the microcapsule shell and *Azolla* cell wall. The microencapsulation process by ionic gelation can provide safe protection for the encapsulated material against high temperatures inside the environment, mainly because of the thin wall that protects the adherent or imprisoned material [37]. Furthermore, the microcapsules that have a complex of phycocyanin-alginate are more stable at high temperatures than free phycocyanin (without encapsulation) [38].

### 3.2. Fresh Macaroni Quality

#### 3.2.1. Chemical Quality Indicators

Table 2 shows the chemical quality metrics (moisture content and water activity) of the fresh macaroni formulations. In respect of moisture content, the four fresh macaroni studied showed no significant variations.

This finding was mostly due to the same quantity of liquid being added to the macaroni formulations. Although having a similar moisture content, the macaroni containing EB and others containing FAP microcapsules had somewhat lower water activity than the CM and macaroni containing FAP.

The reduced water activity values of macaroni containing EB or FAP microcapsules are potentially due to lyophilisation of the microspheres, rendering them and the components drier when compared to the other components used in the CM. Furthermore, alginate and calcium chloride result in an increased water-molecule binding rate, which explains the water activity decrement. Because the formulations’ water activity values permitted bacterial and fungal development, microbiological control was essential throughout preparation and packing [39]. The microbiological quality results of fresh macaroni (data not given) demonstrated conformity with current Egyptian legal microbiological standards, No. 286-1/2005 [40].

#### 3.2.2. Colour Evaluation

The colour of fresh macaroni is a significant quality characteristic that has a major impact on consumer acceptance because it is the first quality characteristic the consumer assesses when purchasing the product [41]. It is based on the properties of the raw ingredient, as well as the manner of processing [42]. The colour of the macaroni was altered by the raw materials used in this investigation (FAP or FAP microcapsules).

Table 3 shows the findings of the colour characteristics of the macaroni samples. The findings demonstrated that fresh macaroni containing EB had a greater L* value, which was related to the white colour of the EB.

Fresh macaroni containing FAP and others containing FAP microcapsules had a green hue, as demonstrated by parameter a*, which can be red (+values) or green (−values), but microencapsulation decreased this green hue propensity (Table 3). If the parameter b* values are positive (+), it shows a preference for yellow, whereas if they are negative (−), it suggests a preference for blue. The b* values were all positive for the produced fresh macaroni (Table 3), showing that the encapsulating agent protected carotenoids and phycocyanin, inhibiting their extraction and bestowing a yellow hue on the fresh macaroni. The abundance of carotenoid pigments may have caused the yellow colour propensity [43]. As a result of the colour of the microalgae, the L*, a*, and b* values of macaroni containing FAP microcapsules and macaroni containing FAP were significantly smaller (*p* ≤ 0.05) than those of the other prepared macaroni. In addition, except for the a* value, there were no significant differences (*p* ≤ 0.05) between macaroni containing FAP microcapsules and macaroni containing FAP in any of the colour characteristics.

#### 3.2.3. Cooking Attributes

For all four fresh spaghetti formulas, five minutes was the ideal cooking time. According to Bastos et al. [44], fresh spaghetti should be cooked for 2 to 4 min. The wheat type used in flour manufacture and pasta preparing operations may have an impact on the timing. Because there is no drying stage with fresh pasta, the cooking time is less than with dried pasta.

When the macaroni formulations were compared with regard to cooking loss (Table 4), it was found that the inclusion of FAP, FAP microcapsules, and EB had no effect on this value, which was under 12%, indicating that the macaroni was of excellent quality [45]. This finding is significant since it is a metric used to assess cooking quality from a consumer and industry perspective [46]. 

The swelling index measures the amount of water absorbed by cooked macaroni, owing to starch gelatinization and protein hydration [47]. The swelling index did not significantly differ (*p* ≤ 0.05) amongst all macaroni formulations, indicating that the substances utilised in bead preparation did not interact with macaroni starch [48]. As a result, starch gelatinization happened in the fresh macaroni in a standard and appropriate manner.

The water absorption values of the macaroni containing FAP and macaroni containing FAP microcapsules were lower (85.93% and 79.85%, respectively) throughout cooking than those of CM, which exhibited the highest value (95.62%) (Table 4).

The inclusion of such non-soluble microparticles into macaroni dough may cause difficulty in water diffusion into the matrix of gluten [49]. Cárdenas-Hernández et al. [50] reported the same finding. Macaroni weight increase values are linked to their gluten content. Gluten’s primary technical role is to construct an internal network that is able to keep the macaroni constituents [51]. The CM and macaroni containing FAP achieved the highest weight increase values (Table 4). This might be owing to the absence of a continuous gluten network along with the alginate presence, resulting in increased starch hydration and an increase in the macaroni weight. The addition of EB and FAP to macaroni may affect its structure by making the gluten network weaker, which is important for retaining amylose throughout the cooking process [47]. Because FAP may have been attached to the inner or outer part of the produced microspheres throughout encapsulation, FAP bound to the beads’ outer shells and impacted the gluten network. Because FAP proteins are unable to form a gluten network, they may act as an impediment to the creation of this network, increasing solid loss.

#### 3.2.4. Fresh Macaroni Texture

Textural characteristics are an important aspect in assuring product acceptability by consumers [52]. Macaroni texture is primarily influenced by its starch matrices, gluten concentration, protein addition, and other components [53]. Firmness is an intriguing characteristic since it is related to the amount of force necessary to crush the macaroni between the teeth [49]. The firmness of the cooked CM increased significantly when it was combined with FAP microcapsules during dough preparation (Table 4), namely from 1.66 ± 0.03 N in the CM to 2.38 ± 0.01 N in the macaroni containing FAP microcapsules. The hardness of the cooked CM was lower than that of the other macaroni formulas. Fradique et al. [46] confirmed the same finding, demonstrating that Chlorella vulgaris and Spirulina maxima biomass incorporation into macaroni led to an increase in their firmness values when compared with the control. Doxastakis et al. [51] confirmed that the cooked fresh macaroni texture is related to the water absorption value throughout the cooking process, which reveals that the FAP microcapsule addition encouraged a greater starch leaching rate, leading to a reduced water absorption capacity throughout the cooking process. As a result, the firmness of the macaroni dough containing FAP microcapsules was enhanced. The macaroni containing FAP also showed starch leaching, smaller water absorption values, and higher cooking loss with cooking time, enhancing the macaroni firmness value. The number of starch granules released from the macaroni dough matrices in the cooking water and coating the macaroni’s surface determines its adhesiveness score [54]. Compared with the CM, all investigated macaroni formulations demonstrated non-significant values of adhesiveness (*p* ≤ 0.05).

#### 3.2.5. Sensory Evaluation

Table 5 presents the sensory evaluation scores of all cooked macaroni (CM and macaroni containing EB, FAP, or FAP microcapsules). Except in texture and colour scores, there were no significant differences (*p* ≤ 0.05) between the macaroni containing FAP microcapsules and CM in all other sensory characteristic scores. In addition, the results in the same table showed that macaroni containing FAP had better sensory scores than CM in all sensory characteristics, with the exception of taste.

Macaroni containing FAP microcapsules had much higher taste and odour scores than macaroni containing FAP, owing to the flavour- and colour-masking functions of microencapsulation [55]. Moreover, the results in Table 5 indicated that the acceptance index of all investigated macaroni formulations after cooking was higher than the recommended value (70%) according to Dutcosky [33]. Based on the sensory test scores, it was found that FAP microcapsules may be used in macaroni formulations without impairing their sensory quality.

## 4. Conclusions

This study was conducted for the purpose of evaluating the effects of adding both free or encapsulated FAP on the quality properties (colour, texture, cooking properties, and sensory properties) of fresh pasta. The produced capsules were evaluated in terms of encapsulation efficiency, solubility, and thermal stability. The FAP microcapsules had irregular sizes with no cracks, high encapsulation efficiency, poor solubility in water, and high thermal stability against water heat treatment. According to the results, FAP microcapsules were perfect for inclusion into fresh macaroni. Macaroni containing FAP microcapsules had somewhat lower moisture content and water activity than the CM. The colour analysis of macaroni containing FAP or FAP microcapsules proved the clear effect of encapsulation, especially on the L* parameter. Moreover, the incorporation of FAP or FAP microcapsules into macaroni had no significant effect on cooking loss or the swelling index. Meanwhile, the water absorbance and weight increase values were decreased. Compared with the CM, macaroni containing FAP microcapsules enhanced the firmness value and demonstrated non-significant values of adhesiveness (*p* ≤ 0.05). The sensory evaluation score indicated that encapsulation could generally increase the customer’s acceptance. As a general conclusion, the findings presented in our study show potential prospects for *Azolla*’s use as a component in fresh macaroni as a healthy dietary option.

## Figures and Tables

**Table 1:** Antioxidant activity of free and microencapsulated *Azolla* before and after heat treatment.

Sample	Unheated *	Heated *
*Azolla*	99.18 ± 1.04 ^Aa^	62.91 ± 1.50 ^Bb^
*Azolla* microsphere	91.26 ± 1.35 ^Ba^	80.49 ± 1.73 ^Ab^

* Equivalent to mmol of trolox/g of *Azolla*. Data are presented as means ± SD. Values with different superscript uppercase letters in a column are significantly different at *p* ≤ 0.05. Values with different superscript lowercase letters in a row are significantly different at *p* ≤ 0.05.

**Table 2:** Chemical quality indicators of the fresh macaroni formulations enriched with free or microencapsulated *Azolla* fern powder.

Parameters	Fresh Macaroni Type			
	CM	Macaroni with EB	Macaroni with FAP Microcapsules	Macaroni with FAP
Moisture (%)	29.88 ± 2.47 ^a^	30.91 ± 2.85 ^a^	31.30 ± 2.06 ^a^	30.72 ± 3.11 ^a^
Water activity	0.97 ± 0.001 ^a^	0.90 ± 0.003 ^c^	0.94 ± 0.002 ^b^	0.95 ± 0.004 ^b^

Data are presented as means ± SD. CM, control macaroni; EB, empty beads; FAP, free *Azolla* fern powder. Values with different superscript lowercase letters in a row are significantly different at *p* ≤ 0.05.

**Table 3:** Colour parameters of the fresh macaroni formulations enriched with free or microencapsulated *Azolla* fern powder.

Parameters	Fresh Macaroni Type			
	CM	Macaroni with EB	Macaroni with FAP Microcapsules	Macaroni with FAP
L*	61.75 ± 0.82 ^b^	64.98 ± 0.71 ^a^	33.18 ± 0.65 ^c^	31.07 ± 0.59 ^c^
a*	1.17 ± 0.06 ^a^	0.95 ± 0.05 ^a^	−5.61 ± 0.09 ^b^	−8.49 ± 1.03 ^c^
b*	16.02 ± 0.88 ^a^	14.73 ± 0.86 ^b^	6.62 ± 0.34 ^c^	5.97 ± 0.54 ^c^

Data are presented as means ± SD. CM, control macaroni; EB, empty beads; FAP, free *Azolla* fern powder. Values with different superscript lowercase letters in a row are significantly different at *p* ≤ 0.05.

**Table 4:** Cooking and textural properties of the fresh macaroni formulations enriched with free or microencapsulated *Azolla* fern powder.

Parameters	Fresh Macaroni Type			
	CM	Macaroni with EB	Macaroni with FAP Microcapsules	Macaroni with FAP
Cooking loss (%)	7.56 ± 0.29 ^a^	8.10 ± 0.42 ^a^	8.53 ± 0.39 ^a^	8.29 ± 0.50 ^a^
Swelling index (%)	1.23 ± 0.02 ^a^	1.68 ± 0.03 ^a^	1.64 ± 0.02 ^a^	1.58 ± 0.04 ^a^
Water absorption (%)	95.62 ± 1.08 ^a^	82.40 ± 1.11 ^b^	79.85 ± 1.16 ^b^	85.93 ± 1.07 ^ab^
Weight increase (%)	19.33 ± 1.32 ^a^	18.23 ± 1.48 ^b^	17.86 ± 1.19 ^b^	18.60 ± 1.20 ^ab^
Firmness (N)	1.66 ± 0.03 ^c^	1.85 ± 0.04 ^b^	2.38 ± 0.01 ^a^	1.92 ± 0.03 ^b^
Adhesiveness (N)	2.88 ± 0.25 ^a^	3.10 ± 0.29 ^a^	2.89 ± 0.18 ^a^	2.71 ± 0.34 ^a^

Data are presented as means ± SD. CM, control macaroni; EB, empty beads; FAP, free *Azolla* fern powder. Values with different superscript lowercase letters in a row are significantly different at *p* ≤ 0.05.

**Table 5:** Sensory evaluation scores of the fresh macaroni formulations enriched with free or microencapsulated *Azolla* fern powder.

Parameters	Fresh Macaroni Type			
	CM	Macaroni with EB	Macaroni with FAP Microcapsules	Macaroni with FAP
Colour	7.71 ± 0.93 ^b^	7.18 ± 1.04 ^b^	8.23 ± 0.39 ^a^	8.07 ± 0.66 ^ab^
Taste	7.42 ± 1.18 ^a^	7.11 ± 1.20 ^a^	7.30 ± 0.95 ^a^	7.27 ± 1.13 ^a^
Texture	7.81 ± 1.14 ^b^	7.39 ± 0.99 ^bc^	8.60 ± 0.12 ^a^	7.88 ± 1.09 ^ab^
Odour	8.56 ± 0.24 ^a^	7.98 ± 0.67 ^b^	8.43 ± 0.30 ^a^	7.04 ± 0.97 ^c^
Overall acceptability	7.88 ± 0.87 ^a^	7.42 ± 0.98 ^b^	8.14 ± 0.44 ^a^	7.57 ± 0.96 ^b^
Acceptance index (%)	87.56 ^b^	82.44 ^c^	90.44 ^a^	84.11 ^bc^

Data are presented as means ± SD. CM, control macaroni; EB, empty beads; FAP, free *Azolla* fern powder. Values with different superscript lowercase letters in a row are significantly different at *p* ≤ 0.05.

## Data Availability

The authors confirm that the data supporting the findings of this study are available within the article.
